# Understanding Water-Stress Responses in Soybean Using Hydroponics System—A Systems Biology Perspective

**DOI:** 10.3389/fpls.2015.01145

**Published:** 2015-12-21

**Authors:** Prateek Tripathi, Roel C. Rabara, Vladimir Shulaev, Qingxi J. Shen, Paul J. Rushton

**Affiliations:** ^1^Department of Biology and Microbiology, South Dakota State UniversityBrookings, SD, USA; ^2^Department of Biological Sciences, University of North TexasDenton, TX, USA; ^3^School of Life Sciences, University of Nevada Las VegasLas Vegas, NV, USA; ^4^Texas A&M AgriLife Research & Extension CenterDallas, TX, USA

**Keywords:** water-stress, drought, soybean, systems biology, ROS signaling, energy metabolism, water-stress physiology, hydroponics

## Abstract

The deleterious changes in environmental conditions such as water stress bring physiological and biochemical changes in plants, which results in crop loss. Thus, combating water stress is important for crop improvement to manage the needs of growing population. Utilization of hydroponics system in growing plants is questionable to some researchers, as it does not represent an actual field condition. However, trying to address a complex problem like water stress we have to utilize a simpler growing condition like the hydroponics system wherein every input given to the plants can be controlled. With the advent of high-throughput technologies, it is still challenging to address all levels of the genetic machinery whether a gene, protein, metabolite, and promoter. Thus, using a system of reduced complexity like hydroponics can certainly direct us toward the right candidates, if not completely help us to resolve the issue.

## Introduction

Water deficiency has a profound impact on ecological and agricultural systems and is a limiting factor in the initial phase of plant growth and establishment (Rochefort and Woodward, 1992; Shao et al., 2009). This results in stomatal closure and reduced transpiration rates, a decrease in water potential, decrease in photosynthetic activities, accumulation of compatible solutes, synthesis of new proteins, and increase in level of reactive oxygen species (ROS) scavenging compounds like ascorbate, glutathione, alpha-tocopherol among others (Ford, 1984; Hoekstra et al., 2001; Jogaiah et al., 2013; Osakabe et al., 2014). Among various important root traits of the root system growth, plant allometry, and hydraulic conductance show significant changes in response to water stress (Comas et al., 2013). Also, process of pollination, seed quality, and yield components are greatly affected (Alqudah et al., 2011). Thus, combating water stress is important for the improvement of crop varieties. To implement the scientific knowledge about the signaling of drought responses in the field, comprehensive understanding of the signaling cascades at the molecular level is important. Moreover, also to find out the critical events that regulate different aspects involved in the signaling pathway (Tripathi et al., 2014). It is crucial to have a detailed understanding of the key regulatory points that can be modulated to have an effective outcome leading to increased productivity and yield. Hence, it is necessary to elucidate the different molecular aspects along with their agronomical aspects so that strategies to produce a better and appropriate drought tolerant variety can be devised.

## Unique findings in hydroponics and its comparison with soil

In this section, we are presenting a comparative overview of the dataset we generate from hydroponically grown plants with published datasets generated from soil-grown plants. The idea behind this perspective is to have a consensus toward the utilization of hydroponics system in water stress research. We are focusing more on metabolite section with the support of transcriptomic and proteomics data, as it comes last in the order and still requires detail study to help us draw a complete picture of drought.

**Figure 1:**
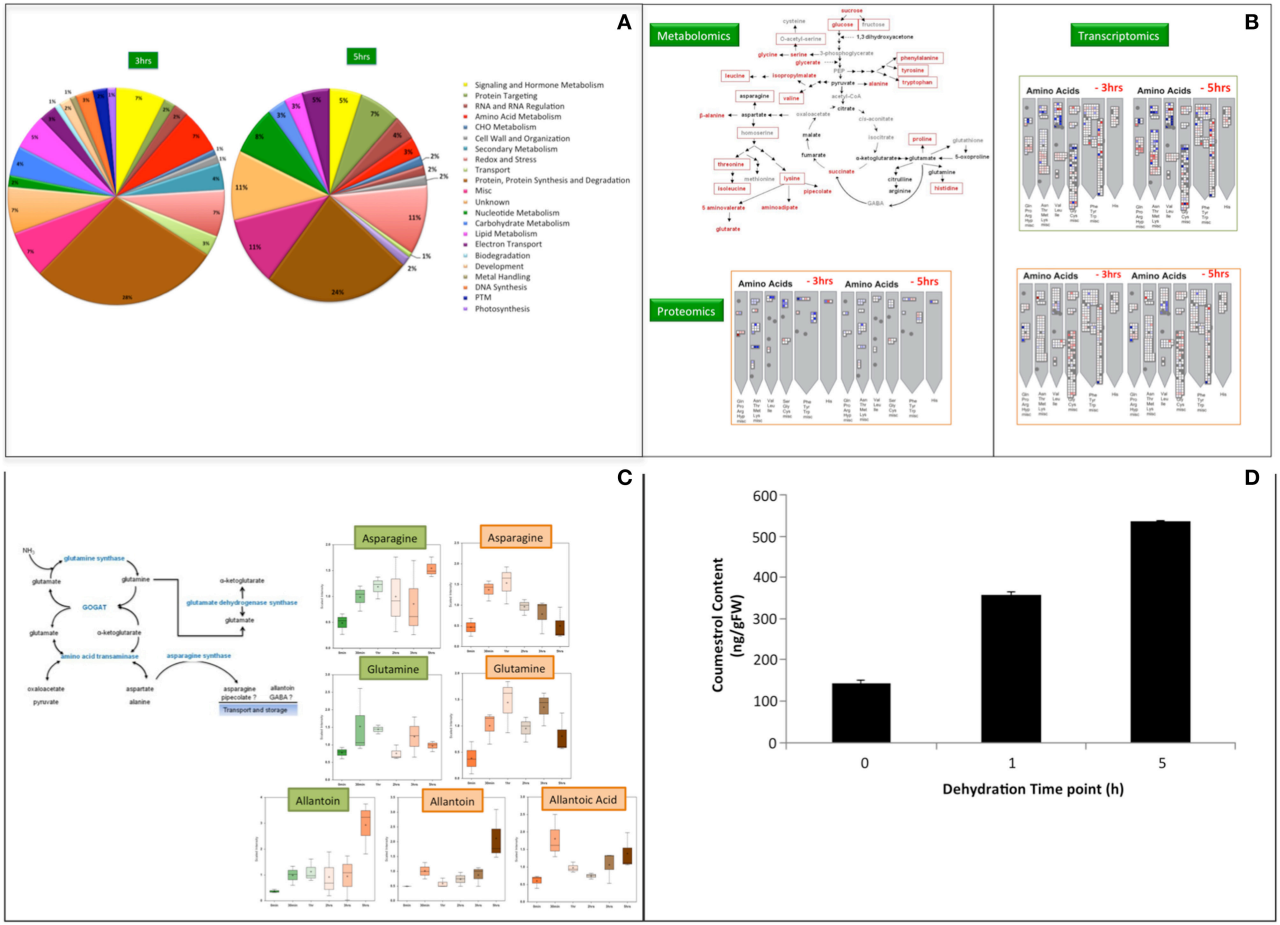
**(A)** Shotgun Proteomics analyses of soybean roots during water deficit. Functional classifications of differentially regulated proteins in soybean samples during water deficit identified by MuDPIT w.r.t 0 min as control. Proteins were functionally classified using Mapman, which is based on the Gene Ontology Consortium and Kyoto Encyclopedia of Genes and Genomes database. Post-translational modification (PTM). The differential regulation was determined with FDR ≤ 5%. **(B)** Systems–wide overview of amino acid elevation during water stress. Metabolomics data at 5h dehydration from root sample is represented in red fonts and from leaves in red boxes. **(C)** Ammonia metabolism during water stress. Aspargine and Allantoin are the main metabolites for nitrogen storage and transport. Allantoic acid was highly abundant in roots only. The data points in **(B,C)** were analyzed for their statistical significance using Welch's two sample *t*-test with 0 min as control and *p* = 0.05. The green box represents leaf profile while the brown box is root profile in both figure sections. **(D)** Coumestrol content in roots of soybean plants subjected to varying level of water stress. The error bars represent standard error of the mean (SEM) (*n* = 3) and significance was analyzed with *t*-test, *p* ≤ 0.05 w.r.t to 0 min as control.

We use hydroponics system, the system of reduced complexity for our systems analysis to identify the potential candidates for water-stress tolerance by plants. Three levels of our systems approach were utilized to uncover possible regulators of dehydration or water stress signaling cascades at mRNA, protein and metabolites level. All three systems levels were analyzed from the same biological samples to facilitate direct comparisons among the three omics datasets (Song et al., 2011). Moreover, all three—omics analyses were therefore performed with the samples harvested at same time points as described in Tripathi et al. (2015). The microarray for gene expression analysis was performed at Mogene LLC (St. Louis, MO) while the shotgun proteomics at BioProximity LLC (Chantilly, VA) and metabolomics was done at Metabolon (Durham, NC) facility. The shotgun proteomics approach for protein profiling during water stress responses in soybean provides information leading to new hypotheses concerning various aspects of water stress signaling (Figure 1A). Data from leave samples was not found conclusive statistically, but we observed significant changes in roots especially at 3 and 5 h post-dehydration. In roots, the proteins involved in amino acid metabolism and biosynthetic pathways were found increased in abundance after 3 h of dehydration. This observation is in agreement with both the transcriptomic (Geo Accession Number GSE49537) and metabolite data (Rabara et al., 2015; Tripathi et al., unpublished). Elevation of free amino acids during water stress in our metabolomics data, poses the question as how this process is being regulated. However, it is hard to know whether amino acid accumulation was due to increased biosynthesis or proteolysis or both. Because biosynthesis of precursors or intermediates of stress affected pathway or accumulation of it due to degradation in response to stress will lead to the accumulation. In the present study, an increase in the main precursors of branched-chain amino acids (BCAA) biosynthesis, namely 2-isopropylmalate, 2,3-dihydroxyisovalerate, 3-methyl-2-oxovalerate, and 4-methyl-2-oxopentanoate, were observed. These findings were consistent with previous studies performed in soybean and other plants (Fukutoku and Yamada, 1981; Ranieri et al., 1989; Joshi et al., 2010; Obata and Fernie, 2012). The increase in BCAA was previously reported due to up-regulation of biosynthetic pathways during drought (Ranieri et al., 1989; Urano et al., 2009), and our observations are consistent with these data. Taken together, these results suggest that increase in amino acids in soybean roots during water stress is due to *de novo* synthesis and not due to protein degradation. Thus, observations from proteomics analysis support the metabolomics findings. Metabolomics, proteomics, and transcriptomics reveals that *de novo* biosynthesis of amino acids occurs due to an increase in the protein levels of the biosynthetic enzymes. So, this is ultimately controlled at the transcriptional level as the mRNA levels for these genes also increase (Figure 1B).

The ammonia detoxification observed in the metabolomics study suggests that nitrogen accumulates during water stress from various plausible sources (Figure 1C). An increase in the level of asparagine and glutamine signifies the accumulation of nitrogen in root tissues (Oliver et al., 2011). Interestingly, studies in *Arabidopsis* and *Brassica* during drought and osmotic stress have shown that an increase in proline is directly related to the effect of increasing asparagine levels (Chiang and Dandekar, 1995). The accumulation of asparagine and glutamine facilitates the transport of nitrogen and carbon for the resumption of growth and metabolism upon rehydration in soybean (Martinelli et al., 2007; Oliver et al., 2011). This transport of nutrients and amino acids could also be facilitating the process of seed filling as soybean accumulates large amounts of seed storage proteins. Also, it was observed that the expression of nitrate transporter1.5 (Glyma09g37220.1) in the roots drastically increased at mRNA level between 2- (10-fold) and 3 h (41-fold) of dehydration. It seems plausible plant's mechanism for cost management during stress conditions over energy metabolism, whereby plants break down complex sugars and transport pyruvate and ADP to the mitochondrial matrix. It is well-understood that a decline in starch level is observed due to a decrease in the rate of photosynthesis. Thus, as a result, the ATP/ADP ratio decreases due to less demand for ATP, which leads to initiation of alternate pathways (Ribas-Carbo et al., 2005). This study also follows the same assumption. Taken together, we hypothesize that there is a flow of nitrogen into amino acid metabolism and *de novo* biosynthesis of amino acids during water stress, and this constitutes a potential water stress tolerance strategy in soybean. This finding is consistent with previous studies that compared drought tolerant and drought sensitive varieties of grasses (Oliver et al., 2011).

Water stress responses result in accumulation or depletion of certain metabolites, alteration of enzyme activities and synthesis of stress-specific proteins. There is a preferential synthesis of metabolites and proteins that affect osmoregulation to maintain growth and energy consumption and hence reflect a cost of acquiring tolerance. An increased amount of carbon flow to metabolites such as malate, aspartate, and alanine is concomitant with a reduced flow of other end products (e.g., carbohydrates) (Zagdanska, 1995). It suggests that NADPH is removed from the chloroplast and enables the formation of sucrose on alternative pathway when sugar synthesis in the calvin cycle is restricted. Leaves co-operatively help in the process by their ability to control excess excitation energy under water stress. Hence, the chloroplast mediated signaling network becomes important in managing ATP utilization during the extreme water stress, and probable action of associated proteins helps in the process of regulation to better withstand the stress condition.

Sugars and sugar alcohols have also been observed to accumulate under drought stress to function as osmolytes. In both roots and leaves, the most predominant accumulation of sugars was raffinose and galactinol, in complete agreement with several previous reports (Foito et al., 2009; Moore et al., 2009). The increased level of osmolytes such as proline, mannitol, trehalose, ononitol, and pinitol was also reported earlier (Ford, 1984; Keller and Ludlow, 1993; Silvente et al., 2012). Thus, we can assign these compounds to their associated function during water stress. For instance, proline and sugars help in the unfolding of proteins and stabilizes membranes (Hoekstra et al., 2001; Ozturk and Demir, 2002; van Heerden and Kruger, 2002; Jogaiah et al., 2013). Trehalose helps in replacing the water by providing a hydrogen-bonding surface and maintaining the folded active states of the protein (Crowe et al., 1997; Almeida et al., 2007; Jogaiah et al., 2013). Our study also supports the suggestion of proline as an indicator of the plant water status not a measure of tolerance (Lazcano-Ferrat and Lovatt, 1999) because proline biosynthesis could be affected by stage of the plant development, relative water content (RWC) of leaves and cultivar of soybean (Silvente et al., 2012).

An increase in isoflavonoids was one of the major findings of this study. The two orders of magnitude increase in the level of coumestrol (~117-fold) a phytoalexin that has high estrogenic activity and is known for the antioxidant activity was one of the major novel findings of this study. Coumestrol, has also been shown to be a UV stress indicator and also plays a role in plant-microbe interactions (Morandi et al., 1984; Beggs et al., 1985; Isobe et al., 2001; Lee et al., 2012). To our knowledge, this compound has not been previously reported as an important increasing metabolite in water stress responses. Accumulation of coumestrol in water-stressed soybean was quantified during early (1 h) and late (5 h) time point (Figure 1D). The level of coumestrol is increased by 2.5-and 3.7-fold during early and late time point, respectively. Glycosylated flavonols and hydroquinone were reported to handle dehydration induced partitioning, as these compounds increase fluidity and depress phase transition temperatures of membranes (Hoekstra et al., 2001; Langridge et al., 2006). A role of flavonoids during abiotic stress is well-established (Samanta et al., 2011). The observation of induction of osmolytes (sugars and sugar alcohols) and accumulation of free amino acids along with the support of the transcriptomics and proteomics data suggests that there is drought-induced ROS formation. Coumestrol biosynthesis is derived from diadzein in soybean (Yu et al., 2003). Up-regulation of proteins for enzymes like chalcone synthase and isoflavone reductase which play a significant role in isoflavonoid biosynthesis has been reported in a proteomics study of soybean root tip (Yamaguchi et al., 2010). However, in the present study no significant increase in diadzein was observed. It is expected if increased flux through the pathway results in the end product coumestrol and not the intermediate, diadzein. We, therefore, propose that the observed major increase in coumestrol level is a response to contribute to ROS scavenging that could be signaled from the chloroplast. The limitation of using hydroponics systems becomes visible in studying the impact of the plant-microbe interaction, especially in nitrogen related pathways, which greatly affects the water-stress responses. Coumestrol was reported earlier that it accumulates at a significant level in mycorrhizal soybean roots (Morandi et al., 1984). It was also reported to enhance the mycorrhizal colonization (Xie et al., 1995). Thus, in our opinion such studies are better answered when the experimental system is soil.

Also, we observed changes in physiological responses using hydroponics system. Dehydrated plants were still able to recover and re-grow when re-placed into the hydroponics solution, showing that plants were still viable even when subjected to extreme dehydration (5 h). Various physiological parameters were monitored to gain insight of the response to water stress. In roots, an 11% decrease in total water content (%TWC) from 3 to 5 h of dehydration was observed while a 10% decrease in leaves %TWC from 2 to 5 h was observed (data not shown). This observation is in accordance with the similar trends seen in experiments performed in soil (Harb et al., 2010). The osmotic potential showed a similar trend. In contrast, the stomatal conductance dropped to about one-third of the control level by 30 min and after 2 h, the stomata were essentially closed.

Our dataset has shown that hydroponics system validates itself to be comparable to field conditions as proposed by soil. We agree with the difference in nature of the complexity of field conditions and growth chambers. However, the use of hydroponics in growing plants to understand the molecular mechanism of plant response to water stress is a valid option. Because imposing water stress is less complex and eliminates combinatorial effects brought by heat and other environmental factors, commonly experience in field-grown plants. Based on our findings, we able to hypothesize about the putative role of coumestrol as another stress biomarker in the leguminous crop like soybeans.

## Conclusion

In light of comparative findings of hydroponics system and soil directs that observations be alike. Also, it greatly minimized the effect of abiotic stresses other than water stress as conditions like temperature, relative humidity, and the light regime were constant. Soybean plants were subjected to a rapid and uniform water deficit stress by removing the plants from the hydroponics solution using the pots. With the use of hydroponics, we have also shown that there is a flow of nitrogen into amino acid metabolism and *de novo* biosynthesis of amino acids during water stress, and this constitutes a potential drought tolerance strategy in soybean. Also, identification of the novel metabolite coumestrol could be another biological marker for understanding and making drought/water stress tolerant crop plants. Validation of these hypotheses needs further experimentation.

## Author contributions

PT and PR designed the study and planned the experiment. PT and RR performed the experiments. VS and QS helped in the analysis. PT wrote the manuscript. All authors have read and approved the manuscript.

## Funding

This work is supported by the National Institute of Food and Agriculture, U.S. Department of Agriculture, under award numbers 2008-35100-04519 and 2008-35100-05969. Any opinions, findings, conclusions, or recommendations expressed in this publication are those of the authors and do not necessarily reflect the view of the U.S. Department of Agriculture.

### Conflict of interest statement

The authors declare that the research was conducted in the absence of any commercial or financial relationships that could be construed as a potential conflict of interest.
